# Activation and suppression mechanisms of the NRG1 helper NLRs

**DOI:** 10.1111/jipb.13928

**Published:** 2025-05-09

**Authors:** Yu‐Ru Wang, Ruize Zhang, Daowen Wang, Yong Wang, Zheng Qing Fu

**Affiliations:** ^1^ Department of Plant Pathology, College of Agriculture Guizhou University Guiyang 550025 China; ^2^ Department of Biological Sciences University of South Carolina Columbia 29208 SC USA; ^3^ National Wheat Engineering Research Center, College of Agronomy Henan Agricultural University Zhengzhou 450046 China

## Abstract

This commentary examines two recent papers featuring intriguing discoveries on the molecular processes and structural foundations involved in the activation and suppression of the N‐requirement gene 1 (NRG1) helper nucleotide‐binding leucine‐rich repeat receptor.

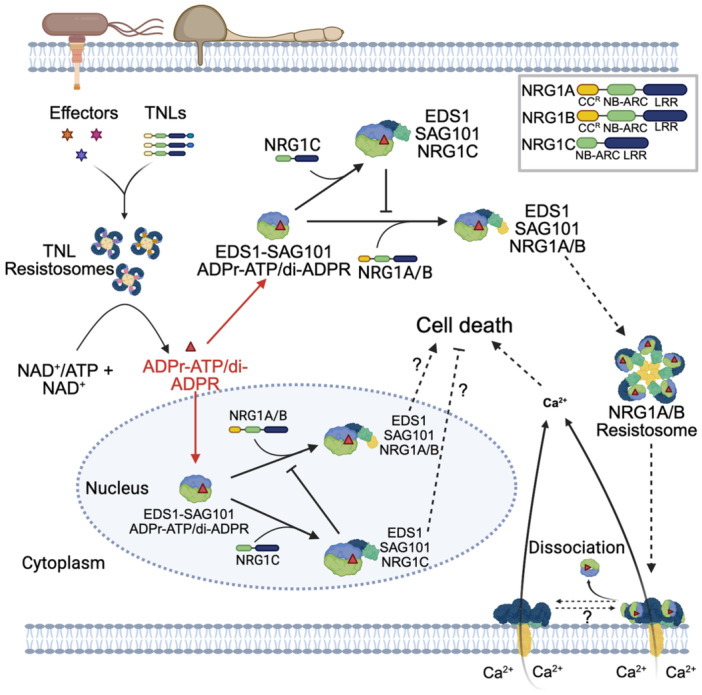

Plants developed a sophisticated defense mechanism to safeguard themselves from a diverse range of pathogens, including fungi, oomycetes, bacteria, viruses, and nematodes. They utilize specific receptors to recognize these threats. Initially, plants rely on pattern recognition receptors located on their cell surfaces to identify conserved molecules referred to as pathogen‐associated molecular patterns (PAMPs), which triggers PAMPs‐triggered immunity (PTI). However, adapted pathogens evolve to deliver effector proteins into host cells to counteract PTI, leading to effector‐triggered susceptibility. In response, plants have acquired intracellular nucleotide‐binding leucine‐rich repeat (NLR) receptors to detect these effector proteins and activate effector‐triggered immunity (ETI) against avirulent pathogens.

Based on their N‐terminal coiled‐coil (CC), Toll/interleukin‐1 receptor (TIR), or resistance to powdery mildew 8‐like (CC^R^) domains, NLRs can be categorized into three families: CC‐NLRs (CNLs), TIR‐NLRs (TNLs), and CC^R^‐NLRs (RNLs). TNLs and CNLs are sensor NLRs responsible for recognizing effectors, whereas RNLs, which include N requirement gene 1 (NRG1) and activated disease resistance 1 (ADR1), act as downstream helper NLRs. When NLRs are engaged in plant defense responses, their actions resemble those of Ne Zha (哪吒), a Chinese mythical and legendary figure with three heads and six arms (三头六臂) (Figure 1A). TNLs, such as RPP1 and ROQ1, oligomerize into tetramers and exhibit NADase activities to catalyze the production of second messengers including adenosine diphosphate‐ribosylated adenosine triphosphate (ADPr‐ATP)/ADPr‐ADPR (di‐ADPR) and 2′‐(5″‐phosphoribosyl)‐5′‐adenosine monophosphate/diphosphate (pRib‐AMP/ADP) (Huang et al., 2023). ADPr‐ATP or di‐ADPR binds to EDS1–SAG101 heterodimer and pRib‐AMP or pRib‐ADP binds to EDS1–PAD4 dimers to activate NRG1 and ADR1, respectively (Figure 1A). CNLs, including ZAR1 and Sr35, oligomerize into pentameric resistosomes, which are then integrated into the plasma membrane to act as Ca^2+^ permeable channels (Jacob et al., 2021; Huang et al., 2023) (Figure 1A). In Arabidopsis plants, alongside the positive regulators NRG1A and NRG1B, there is also an N‐terminally truncated version known as NRG1C (Figure 1B). Unlike NRG1A and NRG1B, NRG1C, when overexpressed, acts as a negative regulator of TNL signaling and can also interact with the EDS1‐SAG101 heterodimer *in planta* (Wu et al., 2022). Despite these remarkable accomplishments, two core questions remain unresolved. First, what is the process by which NRG1 and ADR1 engage with the second messenger‐activated EDS1 heterodimer to activate these helper NLRs? Second, what is the mechanism behind the inhibitory role of NRG1C? Recently, two papers were published consecutively in *Nature*, shedding light on these critical issues (Huang et al., 2025; Xiao et al., 2025).

**Figure 1:**
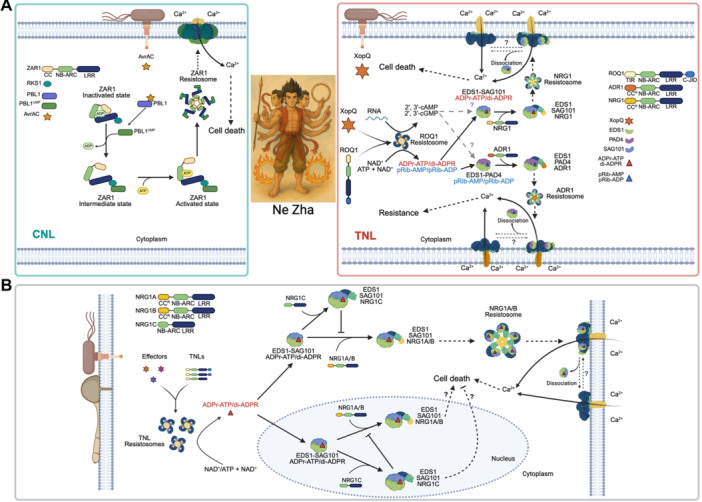
Illustrative diagrams showing the functions of sensor and helper nucleotide‐binding leucine‐rich repeats (NLRs) in effector‐triggered immunity (ETI) and the molecular mechanisms that either activate or inhibit a helper NLR **(A)** Biochemical functions of coiled‐coil (CC) NLRs (CNLs) and Toll/interleukin‐1 receptor (TIR) NLRs (TNLs) and their roles in ETI pathways. The CNL ZAR1 forms a complex with the adaptor protein RKS1 through its C‐terminal LRR (leucine‐rich repeat) domain. When the effector protein AvrAC secreted by pathogenic bacteria modifies the plant kinase PBL1 by converting it to PBL1^UMP^, RKS1 specifically recognizes PBL1^UMP^ and activates ZAR1. ZAR1 undergoes ATP‐dependent oligomerization into a pentameric resistosome. The resistosome's N‐terminal CC domain forms a transmembrane funnel structure that functions as a non‐selective cation channel, mediating Ca²⁺ influx to trigger cell death (membrane targeting mechanism remains unresolved). In the TNL (ROQ1) pathway, direct recognition of the effector XopQ by LRR and C‐JID of ROQ1 induces tetramerization into an NADase‐active resistosome, which hydrolyzes NAD^+^ and ATP to produce second messengers ADPr‐ATP/di‐ADPR and pRib‐AMP/pRib‐ADP. ADPr‐ATP/di‐ADPR binds and allosterically activates the EDS1–SAG101 complex, enabling its interaction with NRG1 to form an oligomeric resistosome. However, its potential dissociation remains uncharacterized. Meanwhile, pRib‐AMP/pRib‐ADP activates the EDS1–PAD4 complex, which recruits ADR1 to form a heterotrimer that induces defense gene expression. Both NRG1 and ADR1 resistosome localize to the plasma membrane as Ca²⁺ channels, elevating cytosolic Ca²⁺ to promote cell death. The assembly of pentameric ZAR1 resistosome and tetrameric ROQ1 resistosome is somewhat reminiscent of Ne Zha, a legendary figure in Chinese mythology known for having three heads and six arms. **(B)** Activation of the NRG1A resistosome and suppression of NRG1A function by NRG1C. Full‐length NRG1A and NRG1B oligomerize into tetrameric resistosomes upon pathogen perception, exhibiting NADase activity that produces ADPr‐ATP/di‐ADPR signaling molecules. These metabolites bind to the EDS1–SAG101 complex, subsequently attracting NRG1A/B to create an EDS1‐SAG101‐NRG1A/B heterotrimer. Structural reorganization of this complex generates a pentameric resistosome with Ca²⁺ channel activity, triggering calcium influx. The truncated isoform NRG1C, lacking the N‐terminal TIR domain, cannot form functional resistosomes but competitively binds EDS1‐SAG101 to suppress NRG1A/B activation in the cytosol and the nucleus, thereby balancing immunity and growth. Dashed outline: nuclear‐localized EDS1‐SAG101‐NRG1A/B heterotrimers (function undetermined). The Ne Zha figure was crafted using AI software ChatGPT, while the remaining parts of the figure were designed with the aid of BioRender (BioRender.com).

Research from both teams showed that when ADPr‐ATP attaches to the EDS1–SAG101 complex, it causes structural alterations in the EP domain of SAG101 (Huang et al., 2025; Xiao et al., 2025). These changes alleviate steric clashes between EDS1‐SAG101 and the WHD‐LRR (winged helix domain – leucine‐rich repeat) region of NRG1A (NRG1A^WHD‐LRR^), allowing ADPr‐ATP‐activated EDS1‐SAG101 heterodimer to interact with NRG1A^WHD‐LRR^. Notably, mutations at key residues on the interaction surface abolished NRG1A activation and effector‐triggered immunity (ETI) responses, highlighting the importance of this recognition step. Interestingly, the way NRG1A is activated is structurally similar to how sensor NLRs like ZAR1 and Sr35 operate (Wang et al., 2019; Zhao et al., 2022). In the typical function of sensor NLRs, the recognition of pathogen effectors initiates structural alterations in the nucleotide‐binding domain (NBD), enabling the exchange of ADP for ATP and leading to allosteric activation (Figure 1A). According to this similarity, Huang and colleagues found that the ADPr‐ATP‐bound EDS1–SAG101 complex interferes with the NBD domain during its interaction with inactive NRG1A. This observation implies that when EDS1‐SAG101 is bound to ADPr‐ATP, it might use a shared allosteric activation method to activate NRG1A monomers, which then oligomerize to form an active resistosome.

Although the EDS1‐SAG101‐NRG1A heterotrimer plays an important role in ETI, the exact process through which ADPr‐ATP‐bound EDS1‐SAG101 aids in the formation of the NRG1A resistosome is still not well understood. One significant obstacle is understanding how ADPr‐ATP‐bound EDS1‐SAG101 assists in the assembly of the NRG1A resistosome. Studies on N‐terminally 16 aa deleted NRG1A variant (NRG1A^∆N16^), which exhibits enhanced oligomerization, suggest that this variant dissociates from EDS1‐SAG101 (Xiao et al., 2025). In contrast, the NRG1A L134E variant, which lacks oligomerization ability, stabilizes the EDS1‐SAG101‐NRG1A heterotrimer. When EDS1‐SAG101 and NRG1A^∆N16^ were co‐expressed in *Nicotiana benthamiana*, western blot analysis detected either EDS1‐SAG101 or NRG1A^∆N16^ oligomers, reinforcing the idea that the heterotrimer likely represents an intermediate state before NRG1A oligomerization. To obtain the heterotrimeric structure *in vitro*, researchers utilized non‐oligomerizing variants such as NRG1A L134E and NRG1A^WHD‐LRR^. Nevertheless, a significant debate persists about whether EDS1‐SAG101 is part of the final NRG1A resistosome and at what point separation happens during oligomerization (Feehan et al., 2023; Wang et al., 2023). Resolving these questions will necessitate additional studies to effectively reassemble the NRG1A resistosome and clarify its assembly mechanism.

NRG1C, which lacks the entire CC^R^ domain and most of the NBD at its N‐terminus, is evolutionarily conserved in Brassicaceae (Wu et al., 2022). It was previously reported that NRG1C interferes with the EDS1–SAG101 complex, fine‐tuning TNL‐mediated immunity (Wu et al., 2022). However, the exact mechanism by which NRG1C inhibits the activation of the NRG1A/B resistosome remains unclear. By reconstituting the EDS1–SAG101–NRG1C complex, both research groups found that NRG1C can interact with the ADPr‐ATP‐bound EDS1–SAG101 complex (Huang et al., 2025; Xiao et al., 2025). In comparison to NRG1A, NRG1C demonstrated a significantly stronger binding affinity for ADPr‐ATP‐bound EDS1‐SAG101, with a dissociation constant (*K*_d_) close to 43 nmol/L, whereas NRG1A's *K*_d_ is roughly 4 μmol/L. Competitive binding assays further confirmed that NRG1C effectively surpasses CC domain deleted NRG1A (NRG1A^∆CC^) for binding to second messenger‐activated EDS1‐SAG101. When the ratio of NRG1C to NRG1A^∆CC^ reached 2, nearly all NRG1A^∆CC^ was outcompeted by NRG1C. EDS1 and SAG101 are both found be nucleocytoplasmic proteins (Wang et al., 2023). NRG1A has been reported to interact with EDS1 in the nucleus when ETI is activated (Feehan et al., 2023). Interestingly, NRG1C is also distributed in both the nucleus and cytoplasm like EDS1 and SAG101 (Figure 1B) (Xiao et al., 2025). Experimental manipulation of its localization, by forcing it to localize to the nucleus, cytoplasm, or plasma membrane, attenuated its ability to block NRG1A‐mediated cell death. This suggests that NRG1C regulates ETI by sequestering the EDS1–SAG101 complex through its nucleocytoplasmic distribution (Figure 1B).

In a manner akin to NRG1C within the NRG1 family, the Arabidopsis ADR1 helper NLR family features a shortened variant known as ADR1‐L3, which is missing the N‐terminal RPW8 domain (Wu et al., 2022). Nevertheless, whereas NRG1C is commonly found across Brassicaceae, an ortholog of ADR1‐L3 has been discovered solely in *Boechera stricta*. Overexpression of ADR1‐L3 does not impair ADR1‐dependent immunity, suggesting that ADR1‐L3 is likely non‐functional. Further research will be required to investigate whether conserved truncated ADR1 clades exist across different plant families and genera. A similar mechanism of immune regulation through suppression is also present in CNL helper NLR systems. An inhibitory pattern has been identified in NRCs (NLR‐REQUIRED FOR CELL DEATH), which serve as helper NLRs (NRC‐H) necessary for various sensor NLRs (NRC‐S) to initiate the hypersensitive response and immunity in in solanaceous plants (Adachi et al., 2023). An atypical NRC‐H clade, NRCX, was identified in five Solanaceae species and was found to suppress cell death caused by autoactive helper NLRs NRC2 and NRC3 in *Nicotiana benthamiana* (Adachi et al., 2023). Notably, silencing NRCX enhances NRC2‐ and NRC3‐dependent cell death, further highlighting its suppressive role in immune signaling.

A key question remains regarding how these inhibitory NRG1C and NRCX benefit plants in restricting pathogen infections. NRG1C and NRCX may exhibit spatiotemporal specificity, potentially fine‐tuning cell death to balance immune responses and growth. While their precise regulatory mechanisms remain unclear, their ability to modulate the hypersensitive response suggests they might help minimize growth costs and play a role in regulating cell death or survival. Further research using spatial and single‐cell technologies could provide deeper insights into their precise roles in regulating plant immunity.

Besides ADPr‐ATP and di‐ADPR, another crucial second messenger in TIR‐mediated cell death is 2′,3′‐cAMP/cGMP, which is generated by TIR domain proteins hydrolyzing double‐stranded RNA (dsRNA) and dsDNA (Figure 1B) (Huang et al., 2023). Loss‐of‐function mutations in Arabidopsis NUDT7, a 2′,3′‐cAMP/cGMP‐specific phosphodiesterase, result in elevated accumulation of 2′,3′‐cAMP/cGMP and stunted growth (Huang et al., 2023). However, this phenotype is suppressed in the *nudt7/eds1‐2* double mutant, suggesting that the production of 2′,3′‐cNMPs by TIRs may act upstream of EDS1. Despite these findings, no direct evidence has shown that 2′,3′‐cAMP/cGMP can bind to the EDS1–SAG101 or EDS1–PAD4 complex. Therefore, further research is needed to elucidate how 2′,3′‐cAMP/cGMP functions in TIR‐dependent immunity and whether it can directly or indirectly induce the formation of the EDS1‐SAG101‐NRG1 heterotrimer, similar to ADPr‐ATP and di‐ADPR.

In summary, these two studies revealed essential molecular processes that both trigger and prevent ETI, significantly advancing our comprehension of plant immunity regulation (Huang et al., 2025; Xiao et al., 2025). The discoveries made in these studies not only deepen our understanding of plant immune responses but could also lead to novel breeding methods to improve disease resistance in crops. However, several questions still need answers. Further investigation is required to determine exactly how the ADPr‐ATP‐bound EDS1–SAG101 complex facilitates the formation of the NRG1 resistosome and to verify if EDS1–SAG101 is an integral part of the functional NRG1 resistosome. Moreover, since both RNLs and CNLs create Ca²⁺‐permeable resistosomes, future research should explore how calcium influx initiates downstream signaling events and eventually results in cell death during immune responses. Lastly, understanding the specific role of inhibitory NLRs, like NRG1C and NRCX, is crucial for uncovering how plants adjust their immune responses. Such knowledge could enable new approaches, like optimizing NRG1A, NRG1B, and/or NRG1C protein levels in the absence and/or presence of pathogens, to precisely control immune signaling, bolstering plant defense without hindering growth.

## CONFLICTS OF INTEREST

The authors declare no conflict of interest.

## AUTHOR CONTRIBUTIONS

Y.R.W., Y. W., and Z.Q.F. conceived the manuscript. The initial draft was created by Y.R.W., Y.W., R.Z., D.W., and Z.Q.F. Subsequent revisions were carried out by R.Z. and Z.Q.F. All authors reviewed and gave their approval to the final version.
